# The effects of resveratrol on the expression of VEGF, TGF-β, and MMP-9 in endometrial stromal cells of women with endometriosis

**DOI:** 10.1038/s41598-021-85512-y

**Published:** 2021-03-15

**Authors:** Tahereh Arablou, Naheed Aryaeian, Sepideh Khodaverdi, Roya Kolahdouz-Mohammadi, Zahra Moradi, Nesa Rashidi, Ali-Akbar Delbandi

**Affiliations:** 1 Department of Nutrition, School of Public Health, Iran University of Medical Sciences, Tehran, Iran; 2 Endometriosis Research Center, Iran University of Medical Science, Tehran, Iran; 3 Department of Immunology, School of Medicine, Iran University of Medical Sciences, Tehran, Iran; 4 Immunology Research Center, Immunology and Infectious Disease Institute, Iran University of Medical Sciences, Tehran, Iran

**Keywords:** Cytokines, Immunological disorders, Immunological disorders, Reproductive disorders, Nutrition

## Abstract

Resveratrol is a phytochemical with anti-angiogenic, anti-inflammatory, and antioxidant properties. The present study has evaluated the effect of resveratrol on the expression of vascular endothelial growth factor (VEGF), transforming growth factor-β (TGF-β) and matrix metalloproteinase-9 (MMP-9) as factors related to endometriosis progression. Thirteen eutopic (EuESCs) and 8 ectopic (EESCs) endometrial stromal cells from women with endometriosis and 11 control endometrial stromal cells (CESCs) were treated with resveratrol (100 µM) for 6, 24 and 48 h. The gene and protein expression levels of VEGF, TGF-β, and MMP-9 were measured using real-time PCR and ELISA methods, respectively. Results showed that the basal gene and protein expression of VEGF and MMP-9 were higher in EESCs compared to EuESCs and CESCs (P < 0.01 to  < 0.001 and P < 0.05 to  < 0.01 respectively). Also, resveratrol treatment decreased the gene and protein expression of VEGF and MMP-9 in EuESCs, EESCs and CESCs (P < 0.05 to  < 0.01 and P < 0.05 to  < 0.01 respectively) and gene and protein expression of TGF-β in EESCs and EuESCs (P < 0.05 to  < 0.01). The effect of resveratrol in reduction of *VEGF* gene expression was statistically more noticeable in EESCs compared to EuESCs and CESCs (P < 0.05). According to the findings, resveratrol may ameliorate endometriosis progression through reducing the expression of VEGF, TGF-β, and MMP-9 in endometrial stromal cells (ESCs).

## Introduction

Phytochemicals are a large group of biologically active compounds found in plants^1^. Intake of dietary phytochemicals has been associated with health benefits and disease prevention^2^.

Resveratrol is a polyphenolic phytochemical belongs to the stilbenoid class^3^. Its main sources are grapes, berries, peanuts, and some other plants^4^. In recent decades, resveratrol is considered because of its beneficiary effects on oxidative stress^5^, inflammation^3^, tumor progression^6^, aging and angiogenesis^7^.

Endometriosis (EM), one of the most prevalent gynecological disorders, is characterized by the growth of endometrial stroma and glands outside the uterine cavity^8^. Its main symptoms are chronic pelvic pain and infertility^9^. To date, several theories have tried to explain the pathogenesis of the disease; among them, Sampson's retrograde menstruation theory is more reputable^10^. This theory stated that translocation of the endometrial cells into the peritoneal cavity through fallopian tubes leads to their adhesion, angiogenesis, and growth in the peritoneum and formation of the ectopic lesions^10,11^. So, according to this theory, growth and angiogenesis factors play vital roles in disease progression.

Vascular endothelial growth factor (VEGF) is shown to play a crucial role in angiogenesis in peritoneal endometriosis. It is secreted by the eutopic endometrium, ectopic endometriotic tissue, and peritoneal fluid (PF) macrophages^12,13^. Previous studies reported the higher concentration of VEGF in PF from patients with endometriosis compared to non-endometriotic controls and its correlation with disease stages^14,15^.

Transforming growth factor-β (TGF-β) is one of the most potent growth factors and monocytes chemoattractants. It can induce fibrosis and angiogenesis in ectopic implants and causes endometriosis progression^16,17^. The PF of women with stage III and IV of endometriosis has higher levels of TGF-β compared to women with milder endometriosis, and a significant decrease in concentrations was achieved after treatment with a gonadotropin-releasing hormone (GnRH) agonist^18^.

Matrix metalloproteinase-9 (MMP-9) is a member of proteinases that plays an essential role in the remodeling of the extracellular matrix^19^. The overexpression of MMP-9 in ectopic endometrial lesions primary seems to exacerbate the angiogenesis and invasion of ectopic implants^20,21^.

It was claimed that resveratrol could ameliorate endometriosis progression^22^. Its effect is due to suppression the expression of growth factors^23^, decrease cell proliferation^24^, reduction of the size of the ectopic implant^25^, induction of apoptosis^26^, reduction of the inflammation^25^ and oxidative stress^27^, and inhibition the invasion, adhesion, and angiogenesis of endometriotic ectopic lesions^22^. Also, Resveratrol has shown to suppress the expression of VEGF and MMP-9 in rat-induced endometriosis^25^ and decrease the expression of VEGF, TGF-β, and MMP-9 in other diseases^28–30^.

Considering the importance of VEGF, TGF-β and MMP-9 in the development of EM and the inhibitory effect of resveratrol on the expression of these factors in other cell types in different diseases, and given that to date no study has been performed on the effect of resveratrol on gene and protein expression of these factors in ESCs, the aim of the present study was to investigate the effect of resveratrol treatment on the gene and protein expression of VEGF, TGF-β and MMP-9 in ectopic (EESCs) and eutopic (EuESCs) endometrial stromal cells in women with endometriosis in comparison with non-endometriotic controls (CESCs).

## Materials and methods

### Study population

The present study was performed on 40 patients with peritoneal endometriosis and 15 non-endometriotic controls. The inclusion criteria were: being at reproductive age (19–45 years old), at the proliferative phase of the menstrual cycle, the III-IV stages of peritoneal endometriosis according to the revised American Fertility Society system (rAFS)^31^ for patients and non-endometriotic lesions for control group based on the laparoscopy. The control group was undergone laparoscopy for diagnostic reasons and because of benign gynecological problems.

The exclusion criteria were: any history of malignancy, autoimmune or metabolic disorders, taking any hormonal medications or dietary supplements within the last three months before the surgery, pregnancy, lactation, and cigarette smoking.

All individuals signed written informed consent before participating in the study, and all participants' privacy was respected. The study protocols was approved by the Ethics Committee for Medical Research of Iran University of Medical Sciences (Code: IR.IUMS.rec.1395.9221324203). All methods were performed in accordance with the relevant guidelines and regulations.

### Sample collection

All sample collection and tissue extraction details were explained earlier^23^. Ectopic and eutopic endometrial samples were collected using laparoscopic sampling and biopsy curette, respectively. All endometriotic cysts (endometrioma) size were ≥ 5 cm in diameter. Tissue samples were put in sterile tubes containing Dulbecco's modified Eagle's medium (DMEM)-F12 (Sigma-Aldrich, St. Louis, MO, USA) culture medium with 1% Penicillin–Streptomycin antibiotics (Gibco, Thermo Fisher Scientific, Waltham, MA, USA) and quickly transferred to the laboratory on ice. A part of all samples was taken to the pathology laboratory to confirm endometriosis. The phase of the menstrual cycle was confirmed by the histological dating of ectopic endometrial implants. In the case of virgins and other patients with only the ectopic tissue, the confirmation of the cycle phase done by the last menstrual period (LMP).

### Isolation, culture and purification of endometrial stromal cells (ESCs)

The digestion of endometrial tissue samples and the purification and culture of stromal cells performed as we described earlier^23^. Briefly, in the sterile condition, ectopic and eutopic endometrial tissues from endometriotic women and normal endometrial tissues from the control women were minced into smaller pieces and digested in the presence of 2 mg/ml Collagenase A (Roche, Pleasanton, CA, USA) and 300 mg/ml DNase (Roche, Pleasanton, CA, USA). Then the obtained cells were cultured in T25 culture flasks (SPL Life Sciences, Korea), ans stored in an atmosphere of 5% CO2 at 37 °C in DMEM-F12 (Sigma-Aldrich, St. Louis, MO, USA) containing 1% Penicillin–Streptomycin antibiotics (Gibco, Thermo Fisher Scientific, Waltham, MA, USA) and 10% fetal bovine serum (FBS) (Gibco, Thermo Fisher Scientific, Waltham, MA, USA) and adherent stromal cells were allowed to multiply. The cultured cells were passaged three times and when they reached to about 80% confluency they were used for the treatment. Some tissue samples especially ectopic tissues were excluded due to the cultural contamination, inproper pathology results, or not obtained the desired cells. At the end, from 40 endometriotic and 15 non-endometriotic control tissues, 8 ectopic, 13 eutopic, and 11 control tissues were treated. The purification of the ESCs was approved by immunofluorescent staining and flow cytometry. These cells were characterized as a panel of vimentin^+^, nestin^+^, cytokeratin^−^, CD10^+^, CD44^+^, CD73^+^, CD105^+^, CD34^−^, and CD45^−^ cells. as we described earlier^23^.

### Treatment of endometrial stromal cells with resveratrol

Based on the results of MTT test and the pilot study^23^, all ESCs from the three study groups were seeded 30 × 10^4^ in 24-well plates (SPL Life Sciences, Korea) to have confluency about 80% for resveratrol treatment. After 3 h treated with the pre-determined optimized concentration of 100 µM resveratrol (Sigma-Aldrich, St. Louis, MO, USA) and stimulated with 100 ng/ml Lipopolysaccharide (LPS) (Sigma-Aldrich, St. Louis, MO, USA)^32^, and incubated for three-time points 6, 24 and 48 h.

### Extraction of RNA and quantitative real-time PCR

For RNA isolation all ESCs were stored in Trizol (Qiagen, Hilden, Germany) at −80 °C. Total RNA was isolated according to the manufacturer’s instructions. Extracted RNA was reverse transcribed to complementary DNA (cDNA) using reverse transcription-polymerase chain reaction (RT-PCR) kit (Fermentas, Thermo Fisher Scientific, Waltham, MA, USA). The gene expressions of *VEGF, TGF-β,* and *MMP-9* were quantified by real-time PCR with Syber premix Extaq (Biofact, Daejeon, Korea) according to the protocol by Rotor-Gene Q (Qiagen, Hilden, Germany). The gene expressions were normalized using Glyceraldehyde 3-phosphate dehydrogenase *(GAPDH)* mRNA as an internal control. The primer pairs and the size of the amplicons are shown in Table 1. The PCR conditions were mentioned in details earlier^23^. It included a holding step on 95° for 15 min (for enzyme activation), which followed by 40 cycles of 95 °C for the 20 s, extension at 60 °C for 40 s (*GAPDH* at 58 °C for 40 s) and the melting step at 60° to 99°. All reactions were run in duplicate.

**Table 1:** The VEGF, TGF-β, MMP-9 and GAPDH primers sequences.

Sequence Name	Accession No	Sequence 5′ to 3'	Amplicon Size (bp)
*VEGF*-Sense	NM_001204384.1 NM_001171622.1	TTGCCTTGCTGCTCTACCTCCA	126
*VEGF*-Anti-sense		GATGGCAGTAGCTGCGCTGATA	
*TGF-β*-Sense	NM_000660.6 XM_011527242.2	TGGTGGAAACCCACAACGAA	113
*TGF-β*-Anti-sense		GAGCAACACGGGTTCAGGTA	
*MMP-9*-Sense	NM_004994.2	GCACGACGTCTTCCAGTACC	124
*MMP-9*- Anti-sense		CAGGATGTCATAGGTCACGTAGC	
*GAPDH*- Sense	NM_001289745.2 NM_002046.6	GCACCGTCAAGGCTGAGAAC	138
*GAPDH*—Anti-sense		TGGTGAAGACGCCAGTGGA	

IGF-1: Insulin-like growth factor-1; HGF: Hepatocyte growth factor; GAPDH: Glyceraldehyde-3-phosphate dehydrogenase; bp: Base pair.

### Measurement of VEGF, TGF-β, and MMP-9 protein

The concentration of VEGF, TGF-β, and MMP-9 protein in the cell supernatant was examined by a standard enzyme-linked immunoassay (ELISA) kit (Duoset; R&D Systems, Minneapolis, MN, USA) according to the manufacturer’s protocol.

### Statistical analysis

Statistical analyses were carried out using GraphPad Prism software 6.01 (GraphPad Software. Inc). Based on the results of the Kolmogorov–Smirnov test, all data was analyzed using the non-parametric tests, including the Wilcoxon sign-ranked test, Mann–Whitney, and Kruskal–Wallis tests. For the gene expression analysis, the fold change and relative expression were compared by calculating the 2^−∆Δct^ and 2^−∆ct^, respectively. P-value < 0.05 was considered as the statistically significant level.

## Results

### The Basal expression of VEGF gene and protein in ESCs

**Figure 1:**
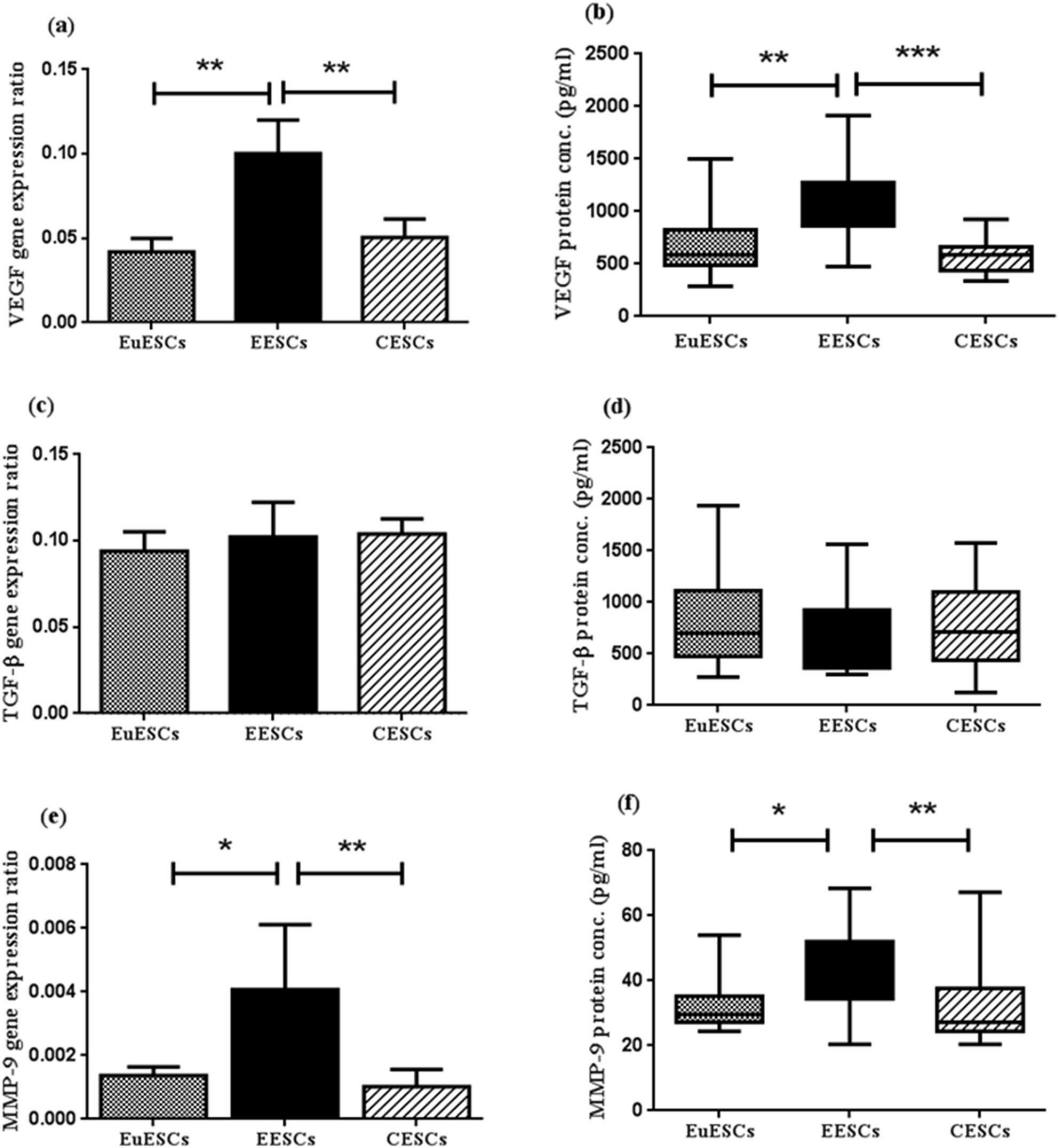
The basal expression levels of VEGF, TGF-β and MMP-9 genes and proteins in ESCs. The basal expression of VEGF, TGF-β and MMP-9 genes and proteins were measured in EESCs (n = 8) and EuESCs (n = 13) from endometriotic women and CESCs from non-endometriotic controls (n = 11) by real-time PCR and ELISA. (**a**) The basal expression of *VEGF* gene, (**b**) The basal expression of VEGF protein, (**c**) The basal expression of *TGF-β* gene, (**d**) The basal expression of TGF-β protein, (**e**) The basal expression of *MMP-9* gene, (**f**) The basal expression of MMP-9 protein. *P-value < 0.05, ** P-value < 0.01 and *** P-value < 0.001 by non-parametric tests. VEGF: Vascular endothelial growth factor, TGF-β: Transforming growth factor-β, MMP-9: Matrix metalloproteinase-9, ESCs: Endometrial stromal cells, EuESCs: Eutopic endometrial stromal cells, EESCs: Ectopic endometrial stromal cells, CESCs: Control endometrial stromal cells.

Based on the results of real-time PCR, the *VEGF* gene was expressed significantly more in EESCs compared to EuESCs and CESCs (Both P < 0.01) in the basic state (Fig. 1a). Moreover, according to the results of ELISA, the VEGF protein had significantly higher expression in EESCs compared to EuESCs and CESCs (P < 0.01 and P < 0.001 respectively) (Fig. 1b).

### The Basal expression of TGF-β gene and protein in ESCs

According to the results of real-time PCR and ELISA the basal gene and protein expression of TGF-β had no statistically significant differences among EESCs, EuESCs and CESCs (Fig. 1c,d).

### The Basal expression of MMP-9 gene and protein in ESCs

Analysis of real-time PCR and ELISA methods revealed that the basal expression of MMP-9 gene and protein was significantly more in EESCs in comparison with EuESCs (Both gene and protein P < 0.05) and CESCs (Both gene and protein P < 0.01) (Fig. 1e,f).

### Resveratrol decreased the expression of *VEGF* gene in all ESCs

The real-time PCR method demonstrated that treatment with resveratrol (100 µM) reduced the expression of *VEGF* gene significantly in EESCs at 24 (P < 0.05) and 48 h (P < 0.01) and in EuESCs and CESCs only at 48 h (Both P < 0.05) (Table 2). Also, the effect of 100 µM resveratrol treatment was more noticeable in EESCs in comparison with EuESCs and CESCs at 48 h (Both P < 0.05) (Supplementary file).

**Table 2:** The effect of resveratrol on *VEGF, TGF-β* and *MMP-9* gene expression in ESCs.

Gene	ESC type	Treatment time	Fold change	P-value
*VEGF*	EuESCs	6 h	0.79 vs 1	0.67
		24 h	0.93 vs 1	0.78
		48 h	0.44 vs 1	0.04*
	EESCs	6 h	0.50 vs 1	0.07
		24 h	0.45 vs 1	0.04*
		48 h	0.19 vs 1	0.007*
	CESCs	6 h	0.99 vs 1	0.64
		24 h	0.72 vs 1	0.99
		48 h	0.43 vs 1	0.04*
*TGF-β*	EuESCs	6 h	1.20 vs 1	0.12
		24 h	1.36 vs 1	0.10
		48 h	0.63 vs 1	0.02*
	EESCs	6 h	0.87 vs 1	0.46
		24 h	0.55 vs 1	0.15
		48 h	0.63 vs 1	0.01*
	CESCs	6 h	1.03 vs 1	0.25
		24 h	1.20 vs 1	0.25
		48 h	1.15 vs 1	0.32
*MMP-9*	EuESCs	6 h	0.53 vs 1	0.49
		24 h	0.20 vs 1	0.002*
		48 h	0.58 vs 1	0.04*
	EESCs	6 h	0.93 vs 1	0.84
		24 h	1.81 vs 1	0.43
		48 h	0.08 vs 1	0.03*
	CESCs	6 h	0.49 vs 1	0.15
		24 h	1.42 vs 1	0.25
		48 h	0.27 vs 1	0.03*

ESCs from endometriotic women (8 EESCs and 13 EuESCs) and non-endometriotic controls (11 CESCs) were cultured with or without 100 µM resveratrol. After 6, 24, and 48 h, the gene expression of *VEGF, TGF-β* and *MMP-9* were examined using real-time PCR. Data were analyzed by non-parametric tests. *P-value < 0.05 is statistically significant.

VEGF: Vascular endothelial growth factor, TGF-β: Transforming growth factor-β, MMP-9: Matrix metalloproteinase-9, ESCs: Endometrial stromal cells, EuESCs: Eutopic endometrial stromal cells, EESCs: Ectopic endometrial stromal cells, CESCs: Control endometrial stromal cells.

### Resveratrol decreased the expression of *TGF-β* gene in EuESCs and EESCs

The *TGF-β* gene expression had significant reduction by treatment with resveratrol (100 µM) in EuESCs and EESCs at 48 h (P < 0.05 and P = 0.01 respectively). The *TGF-β* gene expression had no significant changes in EuESCs and EESCs at 6 and 24 h, and in CESCs at all three time intervals (Table 2). There was no significant difference in the effect of resveratrol treatment between EESCs and EuESCs at 48 h (Supplementary file).

### Resveratrol decreased the expression of *MMP-9* gene in all ESCs

The *MMP-9* gene expression was significantly reduced by resveratrol (100 µM) in EuESCs at 24 (P < 0.01) and 48 h (P < 0.05) and in EESCs and CESCs at 48 h (Both P < 0.05). The gene expression of *MMP-9* did not show significant changes in EuESCs at 6 h, and in EESCs and CESCs at 6 and 24 h (Table 2). In addition, resveratrol had a greater effect on EESCs compared with EuESCs and CESCs at 48 h, but this was not statistically significant (Supplementary file).

### Resveratrol decreased the expression of VEGF protein in EuESCs and EESCs

**Figure 2:**
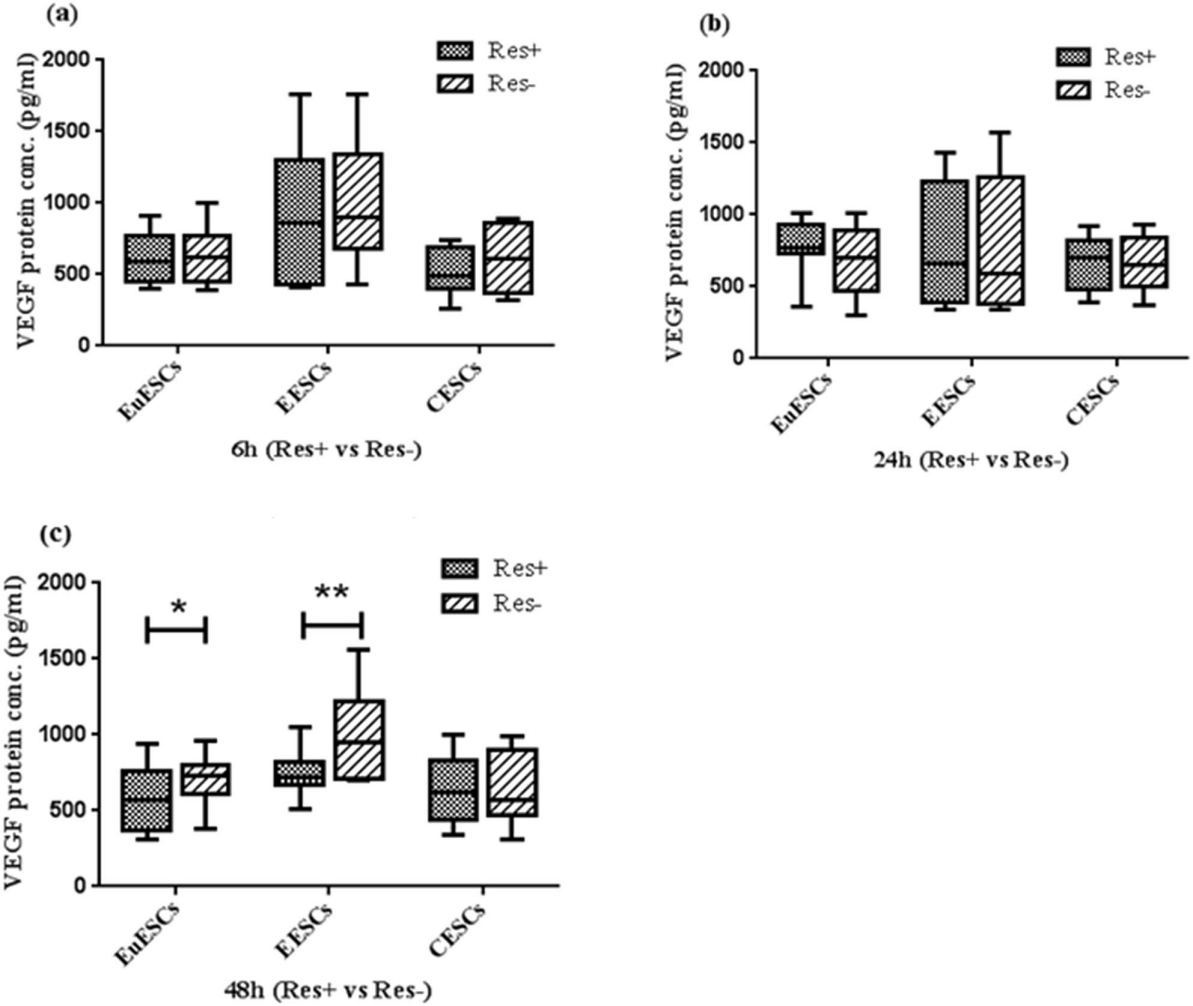
Resveratrol Decreased the Expression of VEGF Protein in EuESCs and EESCs. ESCs from endometriotic women (8 EESCs and 13 EuESCs) and non-endometriotic controls (11 CESCs) were cultured with or without of 100 µM resveratrol. After 6, 24, and 48 h, the protein expression of VEGF was examined using ELISA. (**a**) 6 hr (Res + vs Res-), (**b**) 24 hr (Res + vs Res-), (**c**) 48 hr (Res + vs Res-). *P-value < 0.05, ** P-value < 0.01 by non-parametric tests. VEGF: Vascular endothelial growth factor, EuESCs: Eutopic endometrial stromal cells, EESCs: Ectopic endometrial stromal cells, CESCs: Control endometrial stromal cells.

The use of ELISA method revealed that the protein expression of VEGF was significantly reduced in EuESCs and EESCs at 48 h by 100 µM resveratrol (P < 0.05 and P < 0.01 respectively). Resveratrol treatment had no significant effect at 6 and 24 h in these cells. The VEGF protein expression did not change significantly in CESCs at any of the treatment times (Fig. 2). Although, the effect of resveratrol treatment on reducing VEGF protein expression in EESCs was greater than that of EuESCs, this difference was not statistically significant. (Supplementary file).

### Resveratrol decreased the expression of TGF-β protein in EuESCs and EESCs

**Figure 3:**
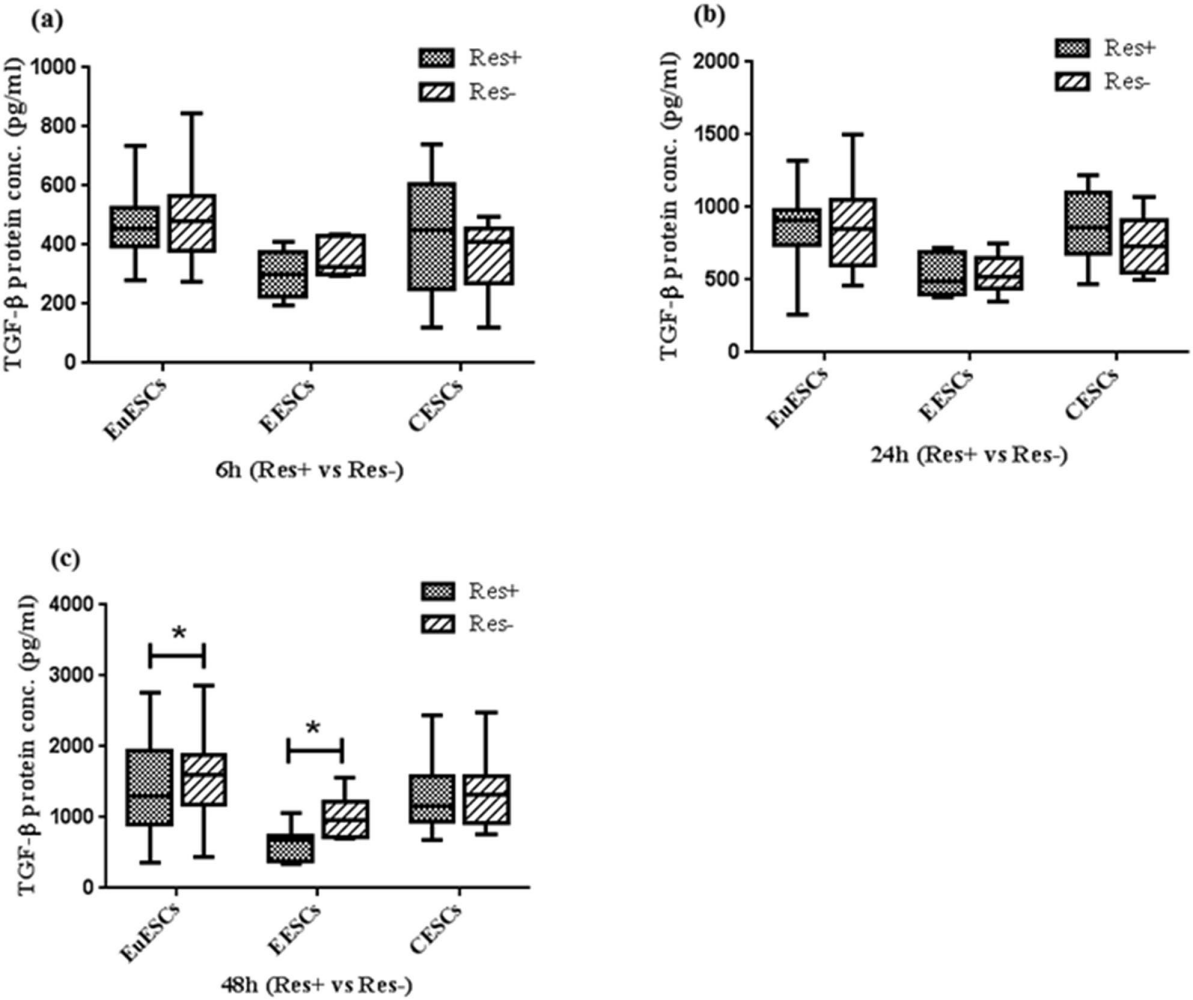
Resveratrol Decreased the Expression of TGF-β Protein in EuESCs and EESCs. ESCs from endometriotic women (8 EESCs and 13 EuESCs) and non-endometriotic controls (11 CESCs) were cultured with or without of 100 µM resveratrol. After 6, 24, and 48 h, the protein expression of TGF-β was examined using ELISA. (**a**) 6 hr (Res + vs Res-), (**b**) 24 hr (Res + vs Res-), (**c**) 48 hr (Res + vs Res-). *P-value < 0.05 by non-parametric tests. TGF-β: Transforming growth factor-β, EuESCs: Eutopic endometrial stromal cells, EESCs: Ectopic endometrial stromal cells, CESCs: Control endometrial stromal cells.

The effect of treatment with 100 µM resveratrol on the expression of TGF-β protein was the same as its gene expression. Resveratrol could reduce the expression of this factor in EuESCs and EESCs at 48 (Both P < 0.05). The expression of TGF-β protein showed no significant changes at 6 and 24 h in EuESCs and EESCs, and at any treatment times in CESCs (Fig. 3). The effect of resveratrol treatment between EESCs and EuESCs had no significant difference at 48 h (Supplementary file).

### Resveratrol decreased the expression of MMP-9 in all ESCs

**Figure 4:**
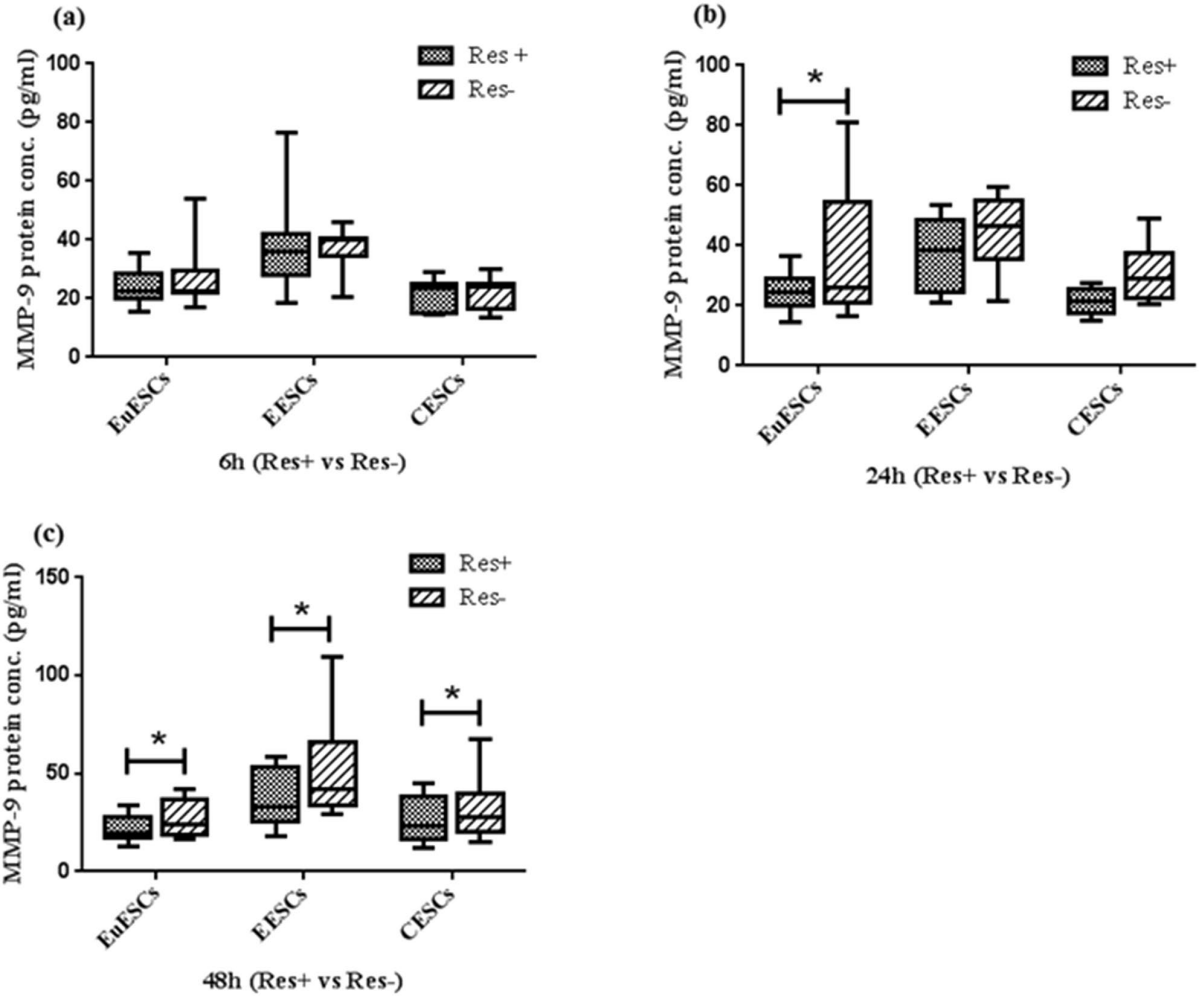
Resveratrol Decreased the Expression of MMP-9 Protein in all ESCs. ESCs from endometriotic women (8 EESCs and 13 EuESCs) and non-endometriotic controls (11 CESCs) were cultured with or without of 100 µM resveratrol. After 6, 24, and 48 h, the protein expression of MMP-9 was examined using ELISA. (**a**) 6 hr (Res + vs Res-), (**b**) 24 hr (Res + vs Res-), (c) 48 hr (Res + vs Res-). *P-value < 0.05 by non-parametric tests. MMP-9: Matrix metalloproteinase-9, EuESCs: Eutopic endometrial stromal cells, EESCs: Ectopic endometrial stromal cells, CESCs: Control endometrial stromal cells.

Resveratrol (100 µM) decreased significantly the MMP-9 protein in EuESCs, at 24 and 48 h and in EESCs and CESCs at 48 h (All P < 0.05). The MMP-9 protein production had no significant changes at 6 in EuESCs at 6 and 24 h in EESCs and CESCs (Fig. 4). In addition, the effect of treatment with 100 µM resveratrol at 48 h on the reduction of MMP-9 protein production was not statistically significant among three groups (Supplementary file).

## Discussion

The results revealed that the basal expression of VEGF and MMP-9, but not TGF-β in EESCs were significantly higher compared to EuESCs and CESCs. To date, some studies assessed the concentration of these factors in PF or endometrial implants of the endometriotic patients^21,33–35^ and according to our knowledge, the present study is the first to compare the expression of VEGF, TGF-β and MMP-9 in EESCs, EuESCs, and CESCs.

The findings of the previous studies, consistent with the present study, have shown that VEGF expression in endometrial tissue and PF of patients with endometriosis is increased compared to controls, although, it does not differ significantly between the different stages of the disease^33,36^. In the only discordant study, the VEGF concentration in PF of patients with genital endometriosis and healthy control women was not significantly different^34^. *VEGF* receptors gene expression was also higher in ectopic endometrial lesions than in eutopic tissue^21,37^.

VEGF is one of the most important angiogenic factors in endometriosis. It can increase cell proliferation, cell migration, and vascular permeability^13,38^. The most important cells secreting this factor in endometriosis are eutopic and ectopic stromal cells, peritoneal macrophages, and neutrophils that increase the expression of this factor in response to elevated inflammatory conditions^13^. Increased levels of reactive oxygen species (ROS) due to oxidative stress in endometriosis can also increase VEGF expression and its angiogenesis in *in-vivo* and *in-vitro*^39^.

The few studies that have examined the expression of TGF-β in endometriosis have shown contradictory findings. For example, in a study of Sokolov et al. the concentration of TGF-β in PF did not differ significantly between the women with genital endometriosis and healthy controls^34^, but two other studies, showed that levels of TGF-β in serum and PF were higher in patients than in controls, and this level, especially in PF, increased with increasing severity of the disease^35,40^.

Several studies have shown the role of TGF-β in regulating the immune system and inflammation^41^. TGF-β enhances the growth and angiogenesis of ESCs, especially ectopic cells, and plays an essential role in the development of endometriotic lesions^13^. Increased expression of this factor in endometriosis seems to occur in response to increased inflammatory conditions and oxidative stress in the peritoneal cavity^42^. As is evident in our study, there was no significant difference in TGF-β expression in the stromal cells of the study groups. Previous studies reported that peritoneal mesothelial cells are the most important source of this factor in peritoneum-related diseases such as peritoneal endometriosis, followed by peritoneal macrophages, ectopic endometrial tissue including ESCs^17^, it appears that the increased expression of this factor in the serum and PF of patients with endometriosis than in controls has been reported in some previous studies^35,40^, may be due to the increased production of this cytokine by peritoneal mesothelial cells and then other sources and in the meantime, the ESCs evaluated in the present study, have less role in the production of this factor. Previous studies have also shown that the concentration of TGF-β in the peritoneum of individuals with endometriosis changes during the menstrual cycle and its highest concentration is seen in the secretory phase and in the premenstrual phase^18,41^. However, in our study, we measured the expression of TGF-β in the proliferative phase.

In the case of MMP-9, the only study comparing the expression of this factor in ectopic endometrial lesions with eutopic endometrium is Machado's study on an induced model of endometriosis in rats and reported findings consistent with the present study^21^.

Studies have shown that chronic inflammation increases MMP-9 expression. Expression of MMP-9 by EESCs and EuESCs increases in endometriosis in response to inflammatory conditions in the peritoneal cavity, which is higher in ectopic than eutopic lesions and activation of the NF-κB and MAP-kinase signaling and other inflammatory pathways, as well as to increased oxidative stress^43,44^; This assists the replacement, growth, and invasion of endometriotic implants^20,21^. Increased production and activity of MMP-9 increase the degradation and regeneration of extracellular matrix, angiogenesis, and VEGF secretion^19,45^.

The present study revealed that the gene and protein expression of VEGF, TGF-β, and MMP-9 in EESCs and EuESCs were reduced by resveratrol treatment. According to our knowledge, this is the first study to investigate the effect of resveratrol on VEGF expression in ESCs of patients with endometriosis. In the only animal study, resveratrol significantly reduced VEGF expression in endometriosis-induced rats^25^. Other previous *in-vivo* and *in-vitro* studies on the effect of resveratrol on VEGF expression in other diseases have also reported findings consistent with the present study^29,46–49^.

The mechanisms of the effect of resveratrol on VEGF expression seems to be through activation of the sirtuin-1 molecule and inhibition of the NF-κB pathway^50^. Resveratrol can also inhibit VEGF through ACE-I-like activity. Thus, resveratrol inhibits positive feedback between angiotensin-II and VEGF. The *in-vitro* studies have shown that ACE-I-like factors can inhibit VEGF-induced endothelial cell migration and invasion and inhibit VEGF mRNA expression^49,51^. Resveratrol may also block the VEGF receptor response pathway by reducing MAP-kinase phosphorylation and inhibiting VEGF-induced angiogenesis by blocking tyrosine phosphorylation in the cadherin molecule^46^. Besides, resveratrol reduces VEGF expression and its invasion and angiogenesis by preventing the production and eliminating the ROS and reactive nitrogen species (RNS)^39,52^.

It seems that the difference in the effect of resveratrol on the reduction of *VEGF* gene expression in EESCs compared to EuESCs and CESCs is due to differences in inflammatory and micro-environmental conditions of these cells. Previous studies have shown that EESCs, EuESCs, and CESCs differ in cytokine expression, cell proliferation, invasion, metastasis, and response to nutritional interventions^53,54^.

The present study is the first to investigate the effect of resveratrol on TGF-β expression in ESCs of patients with endometriosis, and it is not possible to compare the results with similar studies. Therefore, the findings of this study were compared with those of animal studies on the effect of resveratrol on TGF-β levels in other diseases. Most of these studies consistent with the present study have shown that resveratrol can decrease TGF-β gene and protein expression^28,55,56^. In the only inconsistent study, a single-dose intraperitoneal injection of resveratrol had no significant effect on TGF-β levels in rats with acute liver injury, possibly due to the amount and timing of the intervention^57^.

Resveratrol has been reported to inhibit TGF-β transcription by blocking the NF-κB pathway^55^. Resveratrol can also reduce TGF-β expression by blocking the activator protein 1 (AP-1) molecule and removing ROS and reducing oxidative stress^58^. Resveratrol also down-regulates TGF-β expression and activity by down-regulating TGF-β signaling pathway molecules, including, Smad-2, 3,4^59^. TGF-β is a pro-fibrotic factor that can increase the production of type IV collagen and fibrin^28^. Resveratrol treatment can prevent TGF-β-induced fibrotic tissue growth in ectopic lesions^55^.

The present study is the first to assess the effect of resveratrol treatment on MMP-9 expression in ESCs. The only animal study that investigated the effect of resveratrol on MMP-9 expression in endometriosis also reported the same results^25^. Other *in-vivo* and *in-vitro* studies also shown that resveratrol decreases MMP-9 mRNA and protein expression and suppresses the activity of this enzyme^30,60,61^. In the only inconsistent study, Gweon and Kim reported that resveratrol at different concentrations increased the activity and expression of MMP-9 in human fibrosarcoma cells. The cause of this contradictory finding may be the different inflammatory condition^62^.

MMP-9 is one of the proteins whose expression is enhanced by activation of the NF-κB pathway. It appears that resveratrol decreases the expression of this factor by suppressing the expression and activity of the NF-κB pathway^60^. Resveratrol inhibits NF-κB transcriptional activity by blocking phosphorylation and degradation of the IκB inhibitor molecule, thereby inhibiting NF-κB translocation and DNA binding and preventing expression of inflammatory cytokines and growth factors and angiogenesis including MMP-9^63^. Resveratrol can also prevent MMP-9 expression by decreasing TGF-β expression, inhibiting MAP-kinase signaling pathway, reabsorption of ROS, and reducing oxidative stress^64,65^.

The present study had some advantages and limitations: As we mentioned earlier, it was the first study investigated the basal gene and protein expression and also the effect of resverstrol treatment on the gene and protein expression of VEGF, TGF-β and MMP-9 in ectopic (EESCs), and eutopic (EuESCs) endometrial stromal cells of women with endometriosis in comparison with non-endometriotic controls (CESCs). One of the limitations was that the present study was carried out only in the severe (III and IV) stages of the EM and at the proliferative phase. Also, it would have been better if we could assess the MMP-9 activity. It is also better to investigate the effect of resveratrol treatment on the expression of VEGF, TGF-β and MMP-9 in the peritoneal fluid mononuclear cells (PFMCs) and mesothelial cells as the important sources of these factors. Moreover, in order to better determine the effect of resveratrol on EM, further studies are needed on the effect of resveratrol treatment on cell proliferation, angiogenesis, invasion, adhesion, apoptosis, and other processes involved in the pathogenesis of EM.

## Conclusion

The present study showed that the basal gene and protein expression of VEGF and MMP-9 were higher in EESCs compared to EuESCs and CESCs. The treatment of EESCs and EuESCs with resveratrol could reduce the gene and protein expression of VEGF, TGF-β, and MMP-9. Further *in-vitro* and *in-vivo* studies are needed to determine the possible beneficial effects of resveratrol on EM progression.

## Supplementary Information

Supplementary Information

## Data Availability

All data generated or analysed during this study are included in this published article (and its Supplementary Information files).

## Abbreviations

VEGF: Vascular endothelial growth factor
TGF-β: Transforming growth factor-β
MMP-9: Matrix metalloproteinase-9
ESCs: Endometrial stromal cells
EuESCs: Eutopic endometrial stromal cells
EESCs: Ectopic endometrial stromal cells
CESCs: Control endometrial stromal cells
EM: Endometriosis
PF: Peritoneal fluid
GnRH: Gonadotropin-releasing hormone
LMP: Last menstrual period
DMEM: Dulbecco's modified Eagle's medium
LPS: Lipopolysaccharide
RT-PCR: Reverse transcription polymerase chain reaction
ELISA: Enzyme-linked immunoassay
PCR: Polymerase chain reaction
GAPDH: Glyceraldehyde 3-phosphate dehydrogenase
NF-κB: Nuclear factor-κB
MAP-kinase: Mitogen-activated protein kinase
ACE-I: Angiotensin-converting-enzyme inhibitors
ROS: Reactive oxygen species
RNS: Reactive nitrogen species
AP-1: Activator protein 1
