# Supplementary material for: The effects of resveratrol on the expression of VEGF, TGF-β, and MMP-9 in endometrial stromal cells of women with endometriosis
Source: Sci Rep. 2021 Mar 15;11:6054. doi: 10.1038/s41598-021-85512-y (PMC7961000; doi:10.1038/s41598-021-85512-y)
Supplement: Supplementary file 1 — Supplementary Information [file 41598_2021_85512_MOESM1_ESM.pdf]

Supplementary data:

Full title: The effects of resveratrol on the expression of VEGF, TGF-β, and MMP-9 in endometrial stromal cells of women with endometriosis

Short title: The effects of resveratrol on VEGF, TGF-β and MMP-9 expression in endometriosis

Authors: Tahereh Arablou, Naheed Aryaeian, Sepideh Khodaverdi, Roya Kolahdouz-Mohammadi, Zahra Moradi, Nesa Rashidi, Ali-Akbar Delbandi*

-Tahereh Arablou: Department of Nutrition, School of Public Health, Iran University of Medical Sciences, Tehran, Iran. t.arablou@yahoo.com

-Naheed Aryaeian: Department of Nutrition, School of Public Health, Iran University of Medical Sciences, Tehran, Iran. aryaeian.n@iums.ac.ir

-Sepideh Khodaverdi: Endometriosis Research Center, Iran University of Medical Science, Tehran, Iran. khodaverdi.s@iums.ac.ir

-Roya Kolahdouz-Mohammadi: Department of Nutrition, School of Public Health, Iran University of Medical Sciences, Tehran, Iran. roya_kolahdooz@yahoo.com

-Zahra Moradi: Department of Immunology, School of Medicine, Iran University of Medical Sciences, Tehran, Iran. z.moradi1986@yahoo.com

-Nesa Rashidi: Department of Immunology, School of Medicine, Iran University of Medical Sciences, Tehran, Iran. nesarashidi@yahoo.com

-Ali-Akbar Delbandi: Department of Immunology, School of Medicine, Iran University of Medical Sciences, Tehran, Iran AND Immunology Research Center, Immunology and Infectious Disease Institute, Iran University of Medical Sciences, Tehran, Iran. delbandi.ak@iums.ac.ir

***Corresponding Author:** Dr. Ali-Akbar Delbandi, Department of Immunology, School of Medicine, Iran University of Medical Sciences, Hemmat Broadway, Tehran, Iran; Immunology Research Center, Immunology and Infectious Disease Institute, Iran University of Medical Sciences, Hemmat Broadway, Tehran, Iran

Postal code: 1449614535
Postal box: 14665-354
Tel: +982186703287
Fax: +982188622652
E-mail: delbandi.ak@iums.ac.ir

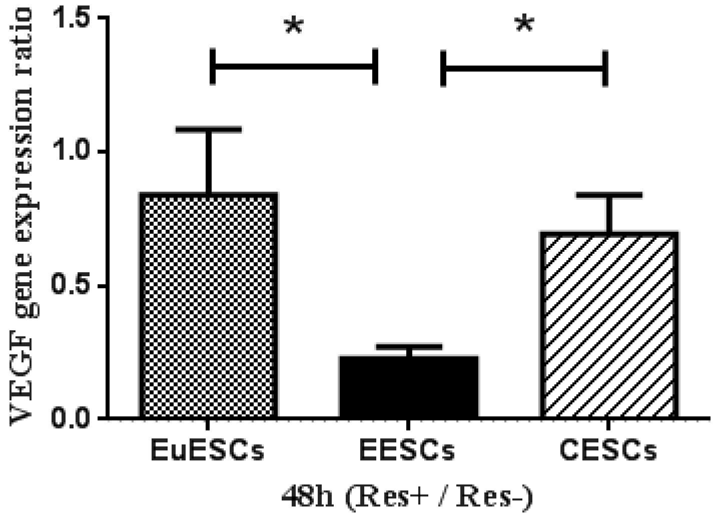

The expression ratio of VEGF genes in ESCs after resveratrol treatment

EESCs (n = 8) and EuESCs (n = 13) from endometriotic patients and CESCs from non-endometriotic controls (n = 11) were cultured in the presence or absence of resveratrol (100μM). The differential effect of resveratrol treatment on gene expression of VEGF was shown as an expression ratio at 48hr. Data were analyzed using non-parametric tests. * P-value <0.05

VEGF: Vascular endothelial growth factor-1, ESCs: endometrial stromal cells, EuESCs: Eutopic endometrial stromal cells, EESCs: Ectopic endometrial stromal cells, CESCs: Control endometrial stromal cells.

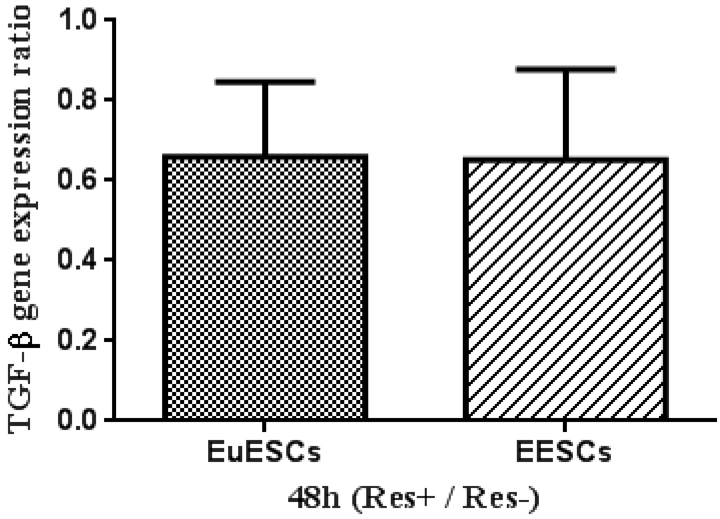

The expression ratio of TGF-β genes between EuESCs and EESCs after resveratrol treatment

EESCs (n = 8) and EuESCs (n = 13) from endometriotic patients and CESCs from non-endometriotic controls (n = 11) were cultured in the presence or absence of resveratrol (100μM). The differential effect of resveratrol treatment on gene expression of TGF-β between EuESCs and EESCs was shown as an expression ratio at 48hr. Data were analyzed using non-parametric tests.

TGF-β: Transforming growth factor-β, EuESCs: Eutopic endometrial stromal cells, EESCs: Ectopic endometrial stromal cells.

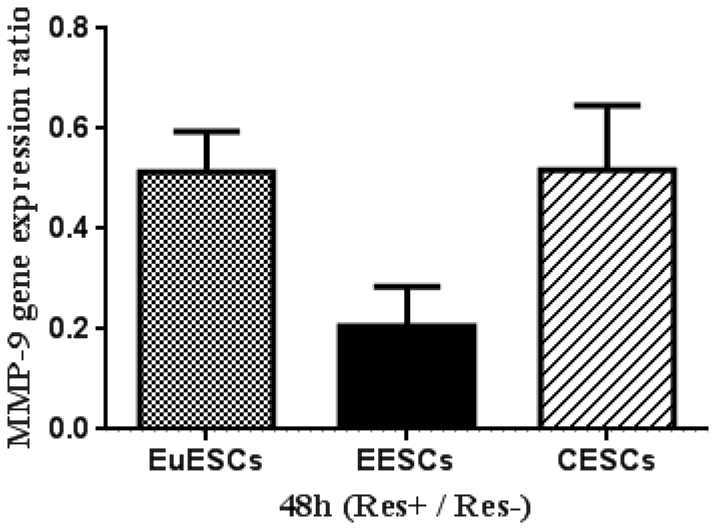

The expression ratio of MMP-9 genes in ESCs after resveratrol treatment

EESCs (n = 8) and EuESCs (n = 13) from endometriotic patients and CESCs from non-endometriotic controls (n = 11) were cultured in the presence or absence of resveratrol (100µM). The differential effect of resveratrol treatment on gene expression of MMP-9 was shown as an expression ratio at 48hr. Data were analyzed using non-parametric tests.

MMP-9: Matrix metalloproteinase-9, ESCs: endometrial stromal cells, EuESCs: Eutopic endometrial stromal cells, EESCs: Ectopic endometrial stromal cells, CESCs: Control endometrial stromal cells.

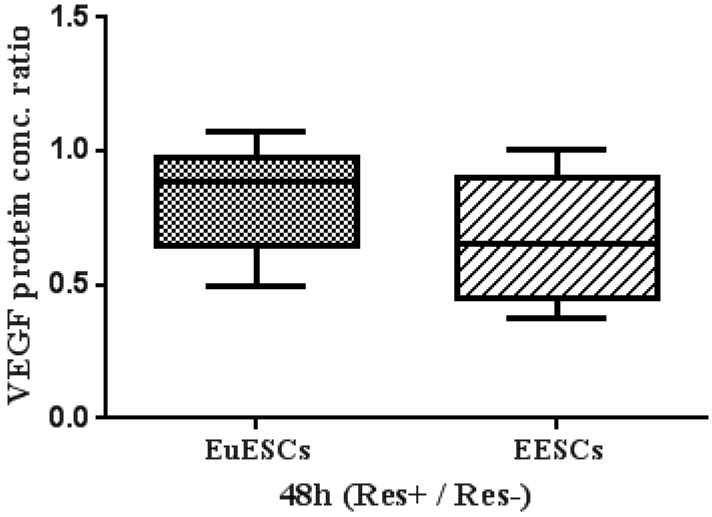

The concentration ratio of VEGF protein between EuESCs and EESCs after resveratrol treatment

EESCs (n = 8) and EuESCs (n = 13) from endometriotic patients and CESCs from non-endometriotic controls (n = 11) were cultured in the presence or absence of resveratrol (100μM). The differential effect of resveratrol treatment on the protein expression of VEGF was shown as the relative expression at 48hr between EuESCs and EESCs. Data were analyzed using non-parametric tests.

VEGF: Vascular endothelial growth factor-1, EuESCs: Eutopic endometrial stromal cells, EESCs: Ectopic endometrial stromal cells.

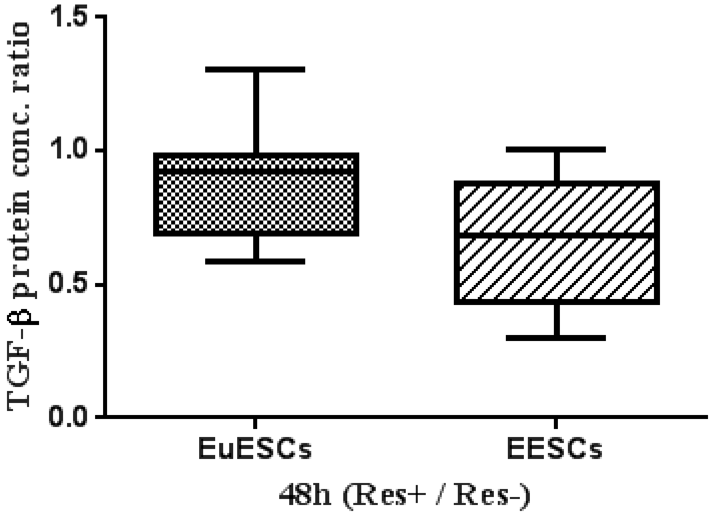

The concentration ratio of TGF-β protein between EuESCs and EESCs after resveratrol treatment

EESCs (n = 8) and EuESCs (n = 13) from endometriotic patients and CESCs from non-endometriotic controls (n = 11) were cultured in the presence or absence of resveratrol (100μM). The differential effect of resveratrol treatment on the protein expression of TGF-β was shown as the relative expression at 48hr between EuESCs and EESCs. Data were analyzed using non-parametric tests.

TGF-β: Transforming growth factor-β, EuESCs: Eutopic endometrial stromal cells, EESCs: Ectopic endometrial stromal cells.

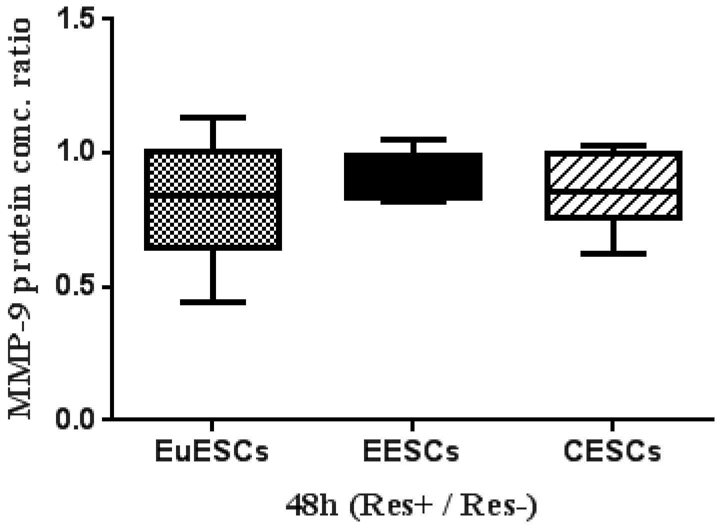

The concentration ratio of MMP-9protein in ESCs after resveratrol treatment

EESCs (n = 8) and EuESCs (n = 13) from endometriotic patients and CESCs from non-endometriotic controls (n = 11) were cultured in the presence or absence of resveratrol (100μM). The differential effect of resveratrol treatment on the protein expression of MMP-9 in ESCs was shown as the relative expression at 48hr. Data were analyzed using non-parametric tests.

MMP-9: Matrix metalloproteinase-9, ESCs: endometrial stromal cells, EuESCs: Eutopic endometrial stromal cells, EESCs: Ectopic endometrial stromal cells, CESCs: Control endometrial stromal cells.
